# Evaluating Machine Learning Models for Classifying Diabetes Using Demographic, Clinical, Lifestyle, Anthropometric, and Environmental Exposure Factors

**DOI:** 10.3390/toxics14010076

**Published:** 2026-01-14

**Authors:** Rifa Tasnia, Emmanuel Obeng-Gyasi

**Affiliations:** 1 Department of Built Environment, North Carolina A&T State University, Greensboro, NC 27411, USA; 2 Environmental Health and Disease Laboratory, North Carolina A&T State University, Greensboro, NC 27411, USA

**Keywords:** machine learning, exposome, multiple imputation (MICE), SMOTE, random forest, environmental exposure, ROC–AUC, predictive modeling

## Abstract

Diabetes develops through a mix of clinical, metabolic, lifestyle, demographic, and environmental factors. Most current classification models focus on traditional biomedical indicators and do not include environmental exposure biomarkers. In this study, we develop and evaluate a supervised machine learning classification framework that integrates heterogeneous demographic, anthropometric, clinical, behavioral, and environmental exposure features to classify physician-diagnosed diabetes using data from the National Health and Nutrition Examination Survey (NHANES). We analyzed NHANES 2017–2018 data for adults aged ≥18 years, addressed missingness using Multiple Imputation by Chained Equations, and corrected class imbalance via the Synthetic Minority Oversampling Technique. Model performance was evaluated using stratified ten-fold cross-validation across eight supervised classifiers: logistic regression, random forest, XGBoost, support vector machine, multilayer perceptron neural network (artificial neural network), k-nearest neighbors, naïve Bayes, and classification tree. Random Forest and XGBoost performed best on the balanced dataset, with ROC AUC values of 0.891 and 0.885, respectively, after imputation and oversampling. Feature importance analysis indicated that age, household income, and waist circumference contributed most strongly to diabetes classification. To assess out-of-sample generalization, we conducted an independent 80/20 hold-out evaluation. XGBoost achieved the highest overall accuracy and F1-score, whereas random forest attained the greatest sensitivity, demonstrating stable performance beyond cross-validation. These results indicate that incorporating environmental exposure biomarkers alongside clinical and metabolic features yields improved classification performance for physician-diagnosed diabetes. The findings support the inclusion of chemical exposure variables in population-level diabetes classification and underscore the value of integrating heterogeneous feature sets in machine learning-based risk stratification.

## 1. Introduction

Diabetes mellitus is a major global public health challenge, contributing to morbidity and mortality worldwide [1]. Global trends indicate a concurrent increase in obesity and diabetes prevalence, with both conditions reflecting complex metabolic dysregulation involving impaired insulin signaling and disrupted glucose homeostasis [2]. In the United States, incidence rates of type 1 diabetes have increased among young adults, with disproportionate growth observed in Hispanic and Black populations [3].

Diabetes risk models have traditionally focused on demographic characteristics (age, sex, race/ethnicity, socioeconomic status) [4], anthropometric indicators (body mass index [BMI], waist circumference, blood pressure), laboratory biomarkers (insulin, lipids, liver enzymes), and behavioral factors such as smoking and alcohol consumption [5,6,7].

Emerging evidence indicates that environmental exposures are associated with diabetes risk. PFAS compounds such as PFOA and PFOS, as well as heavy metals including lead (Pb), cadmium (Cd), and nickel (Ni), have been associated with markers of impaired glucose regulation, insulin resistance, and metabolic dysregulation [8]. Studies increasingly report associations between heavy metal exposures and diabetes prevalence [9,10], with experimental and epidemiologic evidence suggesting potential effects on pancreatic β-cell function and glucose homeostasis [11,12]. With industrialization driving rising environmental levels of heavy metals that frequently exceed safety standards, understanding their role in diabetes-related metabolic dysfunction has become an increasingly urgent public health and analytic priority [13].

Machine learning (ML) has emerged as a valuable analytic approach in population health research. Compared to traditional statistical methods, ML offers enhanced capacity to model complex, nonlinear relationships among biological, environmental, and social determinants of health. Leveraging large, high-resolution datasets, ML techniques can improve disease classification, elucidate exposure–health relationships, and support the development of more precise and effective prevention strategies [14,15,16,17,18,19,20]. Traditional regression-based methods are limited in their capacity to model complex, nonlinear relationships and high-dimensional interactions among risk factors. ML algorithms such as support vector machines (SVMs), artificial neural networks (ANNs), k-nearest neighbors (k-NNs), decision trees, and naïve Bayes offer flexible modeling capabilities that can improve predictive performance. Previous studies applying ML to diabetes risk classification have mainly focused on demographic and clinical predictors, with minimal incorporation of environmental variables [21,22].

KNN is a common and simple method for filling in missing values, but it performs poorly when a large portion of the data is missing [23,24,25]. The challenge becomes more pronounced when datasets contain mixed variable types (nominal, ordinal, binary, and continuous), as these differences can distort underlying data structure and complicate model performance [26,27,28]. Only a limited number of supervised algorithms, primarily tree-based models, can inherently accommodate missing values [29,30]. Earlier KNN-based imputation approaches relied mainly on Euclidean distance and were optimized for continuous data, which often led to misclassification and reduced accuracy when applied to nominal variables or datasets with substantial missingness [31,32,33].

Handling missing data remains a central methodological challenge in population and health research. Multiple imputation has emerged as a practical and robust strategy for reducing bias and preserving statistical power across a wide range of analytic settings [34]. Among available approaches, Multiple Imputation by Chained Equations (MICE), also referred to as Fully Conditional Specification (FCS), is one of the most widely recommended and frequently applied methods [35], which generates multiple plausible values for missing observations through iterative regression models [36,37]. MICE often employs Predictive Mean Matching (PMM), a semi-parametric approach that replaces missing values with observed responses from the most similar cases, making it robust to violations of normality [37].

Missing data in epidemiologic and health studies may arise through several mechanisms with important implications for analytic validity. Missing Completely At Random (MCAR) occurs when the probability of missingness is independent of both observed and unobserved data, often producing a combination of intermittent and monotone missingness patterns [38,39]. More commonly, missingness follows a Missing At Random (MAR) mechanism, in which the likelihood of missingness depends on observed variables but not on the missing values themselves. Both MCAR and MAR can reduce statistical efficiency and introduce bias if not appropriately addressed. MICE is widely recommended for handling missing data under these mechanisms across diverse data structures and study designs [39].

Despite increasing adoption of machine learning approaches in diabetes research, most prior population-based studies have relied primarily on clinical and demographic risk factors, with limited incorporation of environmental exposure indicators. As a result, the potential contribution of toxic environmental exposures to diabetes classification remains insufficiently characterized at the population level. To address this gap, the present study integrates clinical, behavioral, lifestyle, and environmental exposure data within a unified supervised machine learning framework using nationally representative NHANES data. By systematically benchmarking multiple supervised learning algorithms and explicitly evaluating the contribution of environmental toxicants, this work provides a more comprehensive assessment of population-level diabetes classification.

In this study, we leverage nationally representative NHANES 2017–2018 biomonitoring, clinical, behavioral, lifestyle, and environmental exposure data to develop and evaluate an integrated machine learning framework for population-level diabetes classification. Specifically, we pursue the following objectives:

1. To construct a supervised diabetes classification framework that integrates heterogeneous tabular features spanning demographic characteristics, anthropometric measures, clinical laboratory biomarkers, behavioral and lifestyle factors, and toxic environmental exposures.
2. To systematically benchmark multiple supervised classification algorithms, including logistic regression, support vector machines, multilayer perceptrons (artificial neural networks), k-nearest neighbors, decision trees, random forests, naïve Bayes, and XGBoost, under a standardized preprocessing pipeline incorporating feature engineering, multiple imputation, and class-imbalance correction.
3. To identify models that achieve robust out-of-sample generalization and to quantify the relative contribution of toxic environmental exposure features compared with established clinical, behavioral, and lifestyle predictors.

**Figure 1:**
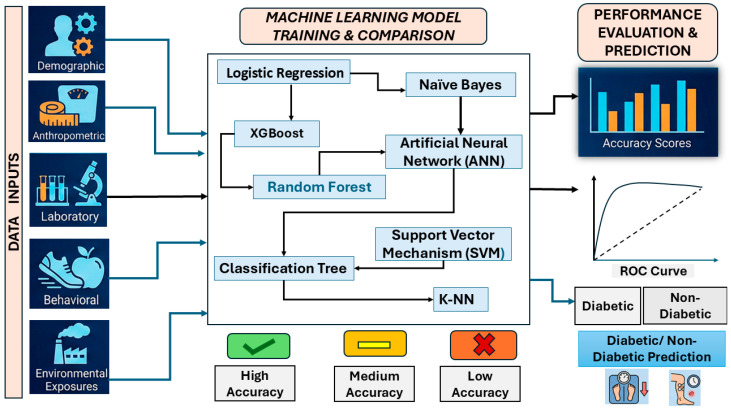
Schematic Diagram of a Multi-Source Machine Learning Pipeline for Diabetes Prediction.

Figure 1 provides an overview of the analytical workflow used in this study, including the data domains incorporated, the supervised machine learning models evaluated, and the procedures used for model training, comparison, and classification.

## 2. Materials and Methods

### 2.1. Data Source and Study Population

NHANES, conducted by the Centers for Disease Control and Prevention through the National Center for Health Statistics, provided the publicly accessible data used in this study. The analytic sample included adults aged 18 years and older with available information across key demographic, socioeconomic, behavioral, anthropometric, clinical, and environmental exposure domains relevant to diabetes risk. Demographic and socioeconomic characteristics included age, sex, race and ethnicity, educational attainment, and household income. Behavioral and lifestyle factors included smoking status and alcohol use. Diabetes status was determined through questionnaire. Anthropometric and clinical measures included body mass index and blood pressure. Environmental toxic exposures were assessed using biomonitoring data for metals and per- and polyfluoroalkyl substances (PFAS).

Because missingness was present in several domains, all variables were harmonized and prepared for multiple imputations to ensure consistent coding and a complete analytic dataset.

### 2.2. Outcome Variable

The primary outcome was self-reported physician-diagnosed diabetes, defined based on the NHANES questionnaire asking whether a doctor or health professional had ever informed the participant of having diabetes. The variable was coded as a binary class:

- 1 = Diabetes (Yes).
- 0 = No diabetes.

### 2.3. Predictor Variables

Predictors included four domains:

1. Demographic variables: age, sex, race/ethnicity, education level, and household income.
2. Behavioral variables: smoking history (≥100 cigarettes), alcohol consumption.
3. Clinical and metabolic biomarkers: insulin, triglycerides, total cholesterol, HDL, LDL, CRP, systolic and diastolic blood pressure, BMI, waist circumference, AST, ALT.
4. Environmental exposure biomarkers: lead, mercury, cadmium, PFOS, PFOA.
5. Composite physiological and behavioral indices: allostatic load and the dietary inflammatory index.

These variables were selected based on clinical relevance, the prior literature, and data availability.

#### 2.3.1. Laboratory Analysis

##### Per- and Polyfluoroalkyl Substances

Serum PFAS were quantified at the CDC National Center for Environmental Health, Division of Laboratory Sciences, Organic Analytical Toxicology Branch using an online solid-phase extraction–high-performance liquid chromatography–TurboIonSpray–tandem mass spectrometry method (online SPE–HPLC–TIS–MS/MS; CDC method 6304.09) in NHANES 2017–2018. Briefly, 50 µL of serum was diluted with formic acid and injected into a commercial column-switching system (Symbiosis extractor with Alias autosampler and HPLC pump; Spark Holland Inc., dba iChrom Solutions, Plainsboro, NJ, USA) that concentrates analytes on an SPE cartridge and then performs reversed-phase HPLC separation. Detection and quantification of target PFAS (including linear and branched PFOA and PFOS isomers, PFNA, PFDA, PFUnDA, PFHxS, GenX, ADONA, and 9Cl-PFESA) were carried out on Sciex Triple Quad 5500, Triple Quad 6500+ or QTRAP 6500 triple-quadrupole mass spectrometers (SCIEX, Foster City, CA, USA) operated in negative-ion TurboIonSpray mode with multiple-reaction monitoring. Instrument control and data acquisition used Analyst software (1.7.4), and peak integration and quantification used MultiQuant (SCIEX, Foster City, CA, USA). Calibration curves were prepared in calf serum using isotopically labeled PFAS as internal standards, and multi-level quality-control pools were analyzed with each batch to monitor accuracy, precision, and long-term assay performance according to CDC laboratory protocols.

##### Blood Metals

Whole-blood cadmium (Cd), lead (Pb), total mercury (Hg), manganese (Mn), and selenium (Se) were measured in NHANES 2017–2018 at the CDC Division of Laboratory Sciences, Inorganic and Radiation Analytical Toxicology Branch, using blood multi-element analysis by inductively coupled plasma–dynamic reaction cell–mass spectrometry (ICP-DRC-MS; CDC method 3016.8). After thorough mixing, EDTA-anticoagulated whole blood was diluted 1:50 with a sample diluent containing tetramethylammonium hydroxide (TMAH, 0.4% *v*/*v*), Triton X-100, ammonium pyrrolidinedithiocarbamate, ethanol, and internal standards (rhodium, iridium, and tellurium), which solubilize cellular components, release metals bound to red blood cells, and stabilize aerosol generation. Diluted samples were introduced via a peristaltic pump and autosampler (ESI SC4-DX, Elemental Scientific Inc., Omaha, NE, USA) into a PerkinElmer ELAN DRC II ICP-MS instrument (PerkinElmer, Norwalk, CT, USA). The plasma was operated at approximately 1450 W radio-frequency power with 15 L/min argon plasma gas; ions passed through the interface and a dynamic reaction cell before mass analysis by a quadrupole. Methane or oxygen reaction gases were used in the DRC to reduce polyatomic interferences on Mn, Hg, and Se. Limits of detection were 0.10 µg/L for Cd, 0.07 µg/dL for Pb, 0.99 µg/L for Mn, 0.28 µg/L for total Hg, and 24.48 µg/L for Se; accuracy and precision were verified against NIST SRM 955c and commercial reference materials, and ongoing quality control followed CDC specifications for this method.

#### 2.3.2. Clinical Analysis

Clinical and anthropometric biomarkers were measured according to standardized NHANES 2017–2018 protocols. Insulin was quantified from venous blood using a hexokinase-mediated enzymatic method and a sandwich immunoassay, respectively, while hemoglobin A1c (HbA1c) was assessed using high-performance liquid chromatography following NGSP-certified procedures [NHANES Lab Methods]. Serum triglycerides, total cholesterol, HDL cholesterol, and LDL cholesterol (calculated using the Friedewald equation when appropriate) were measured enzymatically on automated analyzers with CDC quality-control oversight. High-sensitivity C-reactive protein (CRP) was determined using latex-enhanced nephelometry. Liver enzymes, including aspartate aminotransferase (AST) and alanine aminotransferase (ALT), were quantified using kinetic rate methods. Blood pressure was obtained during the physical examination using a calibrated automatic sphygmomanometer after a standardized rest period, with systolic and diastolic values averaged from repeated measurements. Anthropometric measures were collected by trained health technicians: body mass index (BMI) was calculated from measured height and weight, and waist circumference was measured at the iliac crest using a flexible steel tape according to NHANES anthropometry protocols [NHANES Examination Methods]. All assays followed rigorous internal and external quality-assurance procedures mandated by the CDC’s National Center for Health Statistics.

#### 2.3.3. Derived Scores

##### Allostatic Load

Allostatic load was operationalized as a multi-system index capturing dysregulation across cardiovascular, metabolic, and inflammatory pathways, following established epidemiologic protocols. Allostatic load is the long-term ‘wear and tear’ on the body caused by ongoing stress and repeated adjustments to environmental, social, and metabolic challenges. It has been widely linked to a higher risk of cardiometabolic diseases, including diabetes. Higher allostatic load has been linked to insulin resistance, problems with glucose regulation, and a greater chance of developing type 2 diabetes in studies of large groups of people.

Ten biomarkers were used to construct the score. Cardiovascular indicators included systolic blood pressure (SBP), diastolic blood pressure (DBP), triglycerides, HDL cholesterol, and total cholesterol. Metabolic function was assessed using albumin, body mass index (BMI), hemoglobin A1c (HbA1c), and creatinine clearance. Systemic inflammation was represented by C-reactive protein (CRP).

For each biomarker, values were divided into quartiles based on the distribution within the analytic sample. Participants falling in the highest-risk quartile received a score of 1, and those in lower-risk quartiles received a score of 0. Because lower values of albumin, creatinine clearance, and HDL cholesterol are associated with higher physiological risk, these markers assigned a score of 1 for the lowest quartile instead of the highest. Individual scores across the 10 indicators were summed to create a total allostatic load score ranging from 0 to 10, with higher values indicating greater multisystem physiological strain.

##### Dietary Inflammatory Index

Dietary inflammation was quantified using the Dietary Inflammatory Index (DII), calculated from the 24 h dietary recall interviews administered in NHANES. These recalls were collected using standardized procedures validated by the Nutrition Methodology Working Group. The DII was computed separately for each recall and then averaged to generate a participant-level score.

The computation followed Hébert’s standardized algorithm, which evaluates the inflammatory potential of dietary intake by assessing the contribution of multiple nutrients and food components. In NHANES 2017–2018, data were available for 27 dietary parameters used to derive the DII. These included macronutrients (energy, carbohydrates, protein, fiber), lipid components (saturated, monounsaturated, and polyunsaturated fatty acids), cholesterol, alcohol, and a range of vitamins and minerals (e.g., vitamin A, C, D, E, B6, B12, thiamine, riboflavin, folate, beta-carotene, magnesium, zinc, selenium, iron).

Although Hébert’s full DII algorithm incorporates up to 45 food parameters, prior validation studies show that reliable DII estimates can be generated when fewer components are available, as long as core nutrient contributors are represented. Higher DII scores indicate a more pro-inflammatory dietary pattern.

### 2.4. Data Completeness and Missingness Structure

High variability in missingness across variables was found in a thorough analysis of data completeness, suggesting non-random data gaps. PFOS and PFOA had around 78% missing for each. Other biochemical and clinical indicators, including allostatic load, LDL-cholesterol, insulin, triglycerides, and, exhibited missingness between 68 and 70%.

Demographic variables such as age, gender, and race had no missing values. In contrast, socioeconomic and behavioral factors, including income, education, alcohol usage, and smoking, showed considerable amounts of missing data, ranging from 30% to 45%. In all, 27.6% of the dataset had missing values, meaning that 72.4% of the data could be examined. The Data Quality Distribution analysis grouped the variables into four completeness categories: Perfect (no missing data; 9 variables), Good (up to 20% missing; 10 variables), Moderate (20–50% missing; 17 variables), and Poor (more than 50% missing; 7 variables).

This pattern reveals that most variables contain missing data, making frequent imputation important to prevent losing crucial information. According to the Cumulative Missingness Curve, only 32 columns accounted for roughly 80% of all missing values. This indicates that rather than being dispersed equally throughout the sample, the missing data were clustered in particular exposure and biomarker variables. The Missing Data Pattern Analysis also showed repeated groups of variables missing together, suggesting a structured source of missingness, possibly due to differences in laboratory testing or participant response behavior—rather than completely random missingness.

### 2.5. Handling Missing Data

Missing data were addressed using the MICE method to minimize bias and preserve statistical power. To guarantee convergence and stability, 70 imputed datasets (M = 70) were created across 20 rounds of imputation. Rather than using a single finished dataset, machine learning models were trained and assessed independently on each imputed dataset. Model performance metrics were then aggregated across imputations, allowing both within and between-imputation variability to be reflected in the reported results.

By using predictive models appropriate for each type of variable, the iterative procedure preserved multivariate relationships between predictors. After imputation, findings from all datasets were aggregated using Rubin’s technique, providing pooled estimates that accounted for within- and between-imputation variance.

Rather than randomly maximizing iterations, the number of imputations (M = 70) was determined depending on the degree of missingness and the stability of parameter estimates. Simulation studies [40,41] show that using more imputations than the percentage of missing data does not greatly improve accuracy. Since several variables in this dataset had 60–70% missingness, using 70 imputations (M = 70) was both statistically appropriate and efficient.

NHANES laboratory biomarkers are collected using a complex subsample design rather than full-sample testing. Consequently, the high proportion of missing values for PFOS, PFOA, LDL, insulin, and triglycerides reflects planned subsample testing rather than participant nonresponse. Because assignment to NHANES laboratory subsamples is probabilistic and independent of individual health characteristics, the resulting missingness corresponds to a design-based Missing at Random (MAR) mechanism. Multiple imputation is the recommended approach for handling this type of structured subsample missingness and has been widely applied in peer-reviewed NHANES analyses involving laboratory biomarkers with comparable subsample missingness.

### 2.6. Class Imbalance Handling

The dataset showed a strong class imbalance, with only about 10% of respondents having diabetes. To reduce bias caused by this unequal distribution, we applied the Synthetic Minority Oversampling Technique (SMOTE) after completing multiple imputation. SMOTE generates new diabetes cases by combining each minority sample with its nearest neighbors rather than simply duplicating existing records. This approach produces realistic synthetic examples, helps reduce overfitting, and preserves the structure of the original data. Using SMOTE ensured that diabetic and non-diabetic participants were more evenly represented during model training. SMOTE was applied only within each training fold to ensure that no synthetic minority cases were introduced into the test data and to prevent oversampling from propagating imputed values across folds. Although alternative class-balancing approaches exist (e.g., random undersampling or cost-sensitive learning), SMOTE was selected to provide a consistent and widely adopted framework for model comparison; evaluation of alternative balancing strategies is left for future work.

### 2.7. Model Development

The model’s performance was evaluated using a variety of complementary evaluation metrics due to the dataset’s significant class imbalance. Because false positives have less of an impact on public health than failing to identify people with diabetes, sensitivity (recall) was stressed. Regardless of the classification criteria, overall discrimination was evaluated using ROC-AUC. Precision and F1-score were included to characterize the trade-offs between sensitivity and false-positive burden and to avoid over-reliance on accuracy alone.

Eight machine learning models were trained to classify diabetes status:

- Logistic Regression;
- Classification Tree;
- Random Forest;
- XGBoost;
- Support Vector Machine (RBF kernel);
- Artificial Neural Network (MLP);
- K-Nearest Neighbors;
- Naïve Bayes.

To prevent data leakage, imputation and SMOTE oversampling were performed within each training fold of the cross-validation procedure. Here, the test fold remained untouched during preprocessing. Several metabolic predictors, including insulin, and waist circumference, showed moderate to strong correlations. Multicollinearity was handled using model-specific approaches rather than removing features. Tree-based models, such as Random Forest and XGBoost, are naturally robust to correlated predictors. Sensitivity tests that eliminated strongly correlated diagnostic biomarkers, independent hold-out testing, and cross-validation were used to further assess the robustness of the model.

This study benchmarks a suite of supervised learning algorithms that represent the principal modeling paradigms used in contemporary health analytics. Logistic regression provides a discriminative linear classifier that models the log-odds of diabetes as a function of multiple covariates, offering a stable and interpretable baseline that aligns with established epidemiologic risk-estimation frameworks. A single classification tree supplies a transparent, rule-based model of hierarchical splits, although its high variance limits standalone reliability. Ensemble tree methods extend this class: random forests reduce variance through bootstrap aggregation and randomized feature selection, while XGBoost applies gradient-boosted decision trees with explicit regularization, optimized split scoring, and robust handling of structured tabular data. Support vector machines with an RBF kernel provide a margin-maximizing nonlinear classifier capable of capturing complex decision boundaries in heterogeneous clinical and exposure spaces. Both linear and RBF-kernel SVM variants were initially explored, but only the RBF-SVM was retained and reported because it achieved superior discriminatory performance. The linear SVM is described for methodological completeness, even though only the RBF model was retained for final reporting. Multilayer perceptron neural networks (MLP) introduce a flexible representation-learning architecture that can encode nonlinear interactions and multi-scale dependencies across metabolic, behavioral, and environmental predictors. Naïve Bayes functions as a generative probabilistic baseline that scales efficiently despite its conditional-independence assumption. k-Nearest neighbors serve as a nonparametric, instance-based comparator that reflects local geometric structure but degrades in high-dimensional settings. Together, these models span linear, generative, kernel-based, instance-based, and ensemble learning families, enabling a rigorous comparative assessment of predictive performance using integrated demographic, clinical, lifestyle, anthropometric, and environmental toxic exposure features relevant to diabetes.

All analyses were performed in Python 3.14.2 within a Jupyter Notebook 7.5.2 environment. Data preprocessing and management were carried out using pandas 2.3.3 and NumPy 2.4.1, with scikit-learn 1.8.0 utilities supporting feature engineering, standardization, class balancing, and multiple imputation workflows. Model development employed scikit-learn implementations of logistic regression, support vector machines, multilayer perceptrons, k-nearest neighbors, naïve Bayes, classification trees, and random forests. Gradient-boosted models were trained using XGBoost 3.1.3. Hyperparameter selection used grid-based search with cross-validation through GridSearchCV to ensure stable model calibration across imputations. Performance metrics and pooled estimates from the multiply imputed datasets were computed using scikit-learn’s evaluation suite. Visualizations and diagnostic plots were generated with Matplotlib 3.10.8 and Seaborn 0.13.2. The Jupyter environment supported a fully reproducible workflow for iterative experimentation, hyperparameter tuning, and integration of clinical, behavioral, anthropometric, and environmental exposure features.

### 2.8. External-Style Validation (Internal Hold-Out Validation)

An internal hold-out test was conducted for validation. Using stratified sampling to maintain the diabetes/non-diabetes ratio, the entire dataset was split into 80% for model building and 20% for independent testing after multiple imputations were completed. To avoid information leakage, all preprocessing procedures, standardization, SMOTE balancing, and hyperparameter tuning were limited to 80% of the training data. After that, the final Random Forest and XGBoost models were assessed on the unaltered 20% test subset and retrained on the training set. This allowed us to determine how well the models generalized to new data and to provide evidence of external validation.

## 3. Results

### 3.1. Descriptive Statistics

The study sample had an almost equal distribution of sex. Females were 50.76% and males were 49.24%. Income was spread across many categories. Most people were in the middle-income range, especially in the $35,000–$44,999 (15.24%) and $45,000–$54,999 (14.17%) ranges. Very few were in the $10,000–$14,999 group (4.00%).

Regarding lifestyle, 40% of participants smoked at least 100 cigarettes in their lives, and 60% had never smoked. Alcohol use was widespread; 89% reported drinking alcohol, and only 11% did not drink. Only 10% of the sample had diabetes, and 90% did not. Regarding education, 8.64% had less than a 9th-grade education, and 11.50% had a 9th–11th-grade education. High-school graduate or GED was 23.84%. The largest group, 31.99%, had some college or an associate degree. Another 24.04% were college graduates or above.

**Figure 2:**
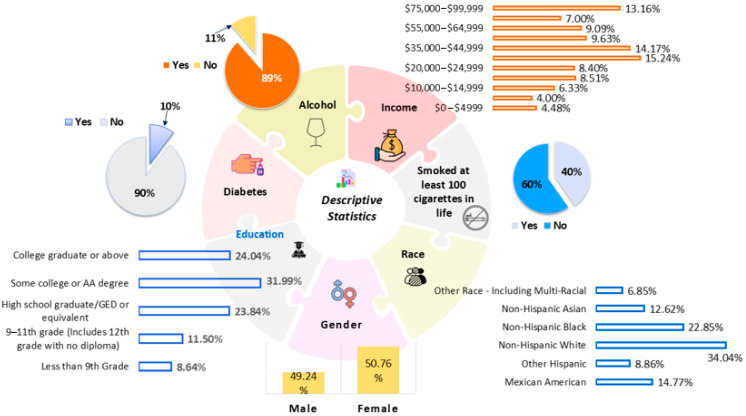
Descriptive statistics of the study participants.

The sample was also racially diverse. Non-Hispanic White participants accounted for 34.04%, and Non-Hispanic Black participants accounted for 22.85%. Mexican Americans were 14.77% and Non-Hispanic Asians were 12.62%. Other Hispanic participants were 8.86%, and 6.85% belonged to other or multiracial groups. Overall, the dataset shows a mixed population with variation in gender, income, education, race/ethnicity, and health-related behaviors. The demographic information can be found in Table 1 and Figure 2.

### 3.2. Missing Data Structure and Imputation Strategy

**Figure 3:**
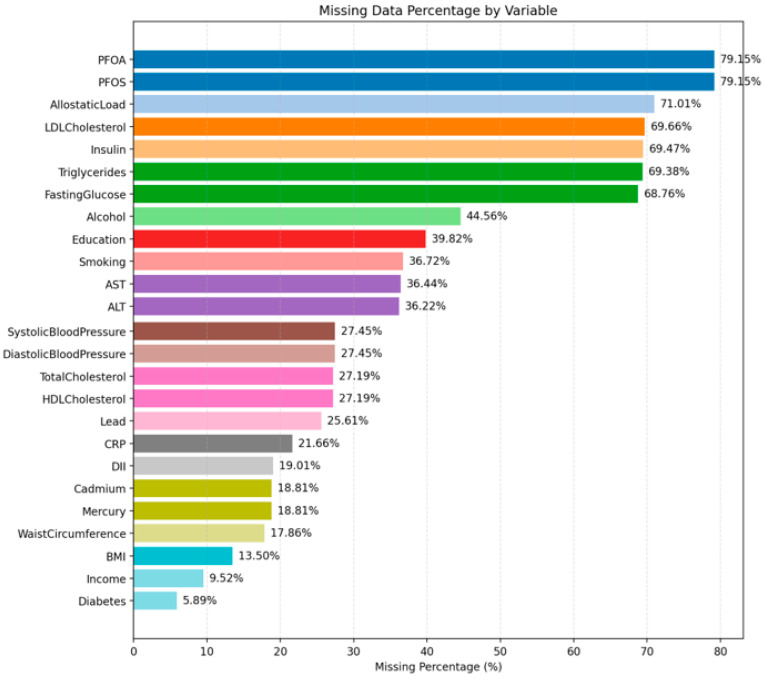
Variable-wise data availability. The bar plot displays variables ordered by missingness, highlighting PFOS and PFOA as the least complete indicators, followed by allostatic load and key metabolic biomarkers.

Figure 3 demonstrates the pronounced heterogeneity in missing data across all variables examined. The most extensive missingness occurs in the PFAS biomarkers PFOA and PFOS, each with roughly 79 percent of observations unavailable. Allostatic load also shows a substantial proportion of missing values at approximately 71 percent. Several metabolic biomarkers, including LDL cholesterol, insulin, and triglycerides follow closely with missingness in the 69 to 70 percent range. Moderate levels of missing data are evident for lifestyle and clinical indicators. Alcohol use exhibits 45 percent missingness, while education, smoking, AST, and ALT fall between 35 and 40 percent. Measures such as systolic and diastolic blood pressure, total cholesterol, HDL cholesterol, and the metal biomarkers show missingness levels near 25 to 27 percent. Conversely, BMI, income, and diabetes status display comparatively low levels of missingness, all below 15 percent.

### 3.3. Machine Learning Model Performance Comparison

**Figure 4:**
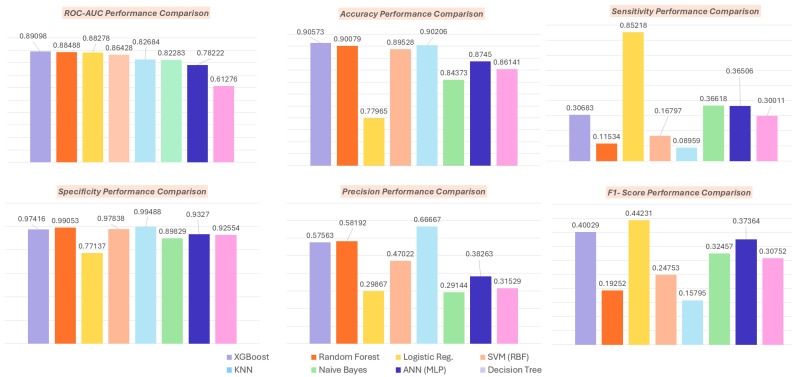
Model performance comparison across evaluation metrics after multiple imputation (M = 70) and SMOTE class balancing. Bars show ROC–AUC, accuracy, sensitivity, specificity, precision, and F1-score for each of the eight machine learning models. XGBoost and Random Forest demonstrate strong overall discrimination and accuracy, while logistic regression achieves the highest sensitivity and F1-score, highlighting trade-offs across performance metrics.

After applying Multiple Imputation by Chained Equations (MICE; M = 70) to address missing data and using the Synthetic Minority Oversampling Technique (SMOTE) to balance the classes. Eight machine learning models were trained and evaluated with stratified 10-fold cross-validation. The pooled performance across all imputations is shown in Table 2 and illustrated in Figure 4. XGBoost delivered the strongest performance across many metrics.

**Figure 5:**
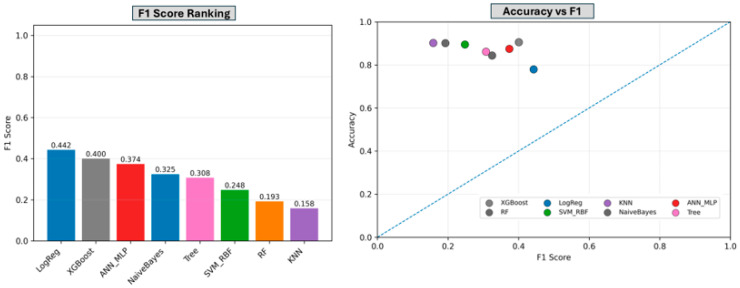
Comparative performance of the machine learning models across two complementary metrics. **Left panel**: Models ranked by F1-score. **Right panel**: Accuracy versus F1-score for each model.

Figure 5 summarizes model performance across complementary evaluation metrics under class imbalance. The left panel ranks classifiers by F1 score, highlighting differences in minority-class discrimination across algorithms. XGBoost and logistic regression achieved the highest F1 scores, followed by naïve Bayes and the multilayer perceptron, indicating superior balance between precision and recall for the positive class. The right panel visualizes the relationship between accuracy and F1 score for each model, illustrating trade-offs between overall correctness and minority-class performance. Collectively, these results demonstrate that high accuracy does not necessarily translate to strong minority-class discrimination, underscoring the importance of multi-metric evaluation for robust and reliable model comparison in imbalanced classification settings.

**Figure 6:**
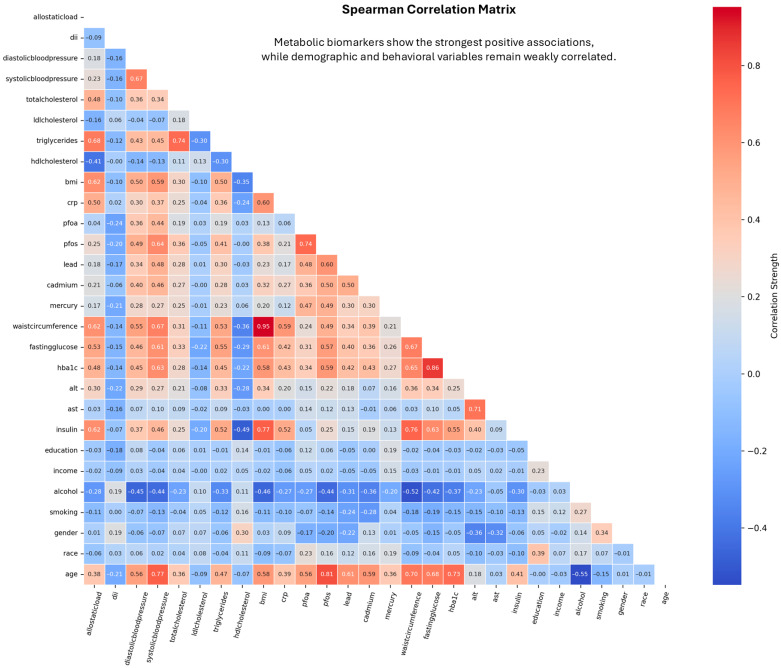
Spearman correlation matrix showing pairwise monotonic associations among demographic, behavioral, anthropometric, clinical, biochemical, and environmental exposure variables. Metabolic biomarkers, including fasting glucose, HbA1c, insulin, triglycerides, LDL cholesterol, BMI, and waist circumference, demonstrate the strongest positive correlations, reflecting shared physiological pathways. Environmental toxicants (lead, cadmium, mercury, PFOA, PFOS) exhibit generally weak correlations with most clinical and behavioral variables, indicating largely independent exposure patterns.

Figure 6 presents a full-matrix Spearman correlation heatmap summarizing pairwise monotonic associations among clinical, biochemical, behavioral, demographic, and environmental exposure features. Fasting glucose and hemoglobin A1c were excluded from all machine learning models because they are components of the diagnostic definition of diabetes. As expected, the strongest positive associations were observed among metabolic biomarkers, reflecting shared physiological pathways related to glucose regulation and lipid metabolism. Environmental exposure biomarkers, including lead, cadmium, mercury, PFOA, and PFOS, displayed limited pairwise monotonic associations with most metabolic and behavioral variables. Behavioral factors such as smoking and alcohol use, along with demographic characteristics including age, sex, race or ethnicity, education, and income, similarly exhibited modest pairwise associations across feature domains, indicating minimal collinearity while preserving the potential for multivariate contributions in downstream modeling.

**Figure 7:**
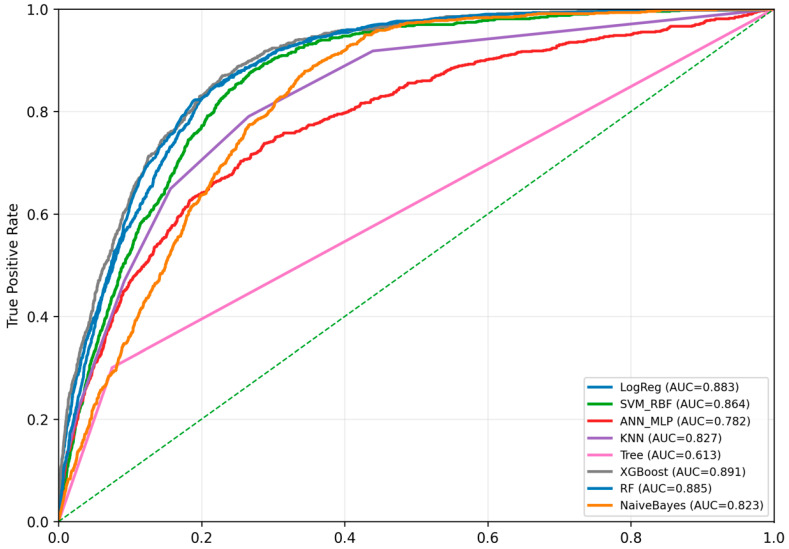
ROC curves for the eight machine learning models trained on the multiply imputed and SMOTE-balanced dataset. XGBoost and Random Forest demonstrate the highest predictive discrimination, with ROC–AUC values of 0.891 and 0.885, respectively. The Support Vector Machine (RBF kernel), Artificial Neural Network, k-Nearest Neighbors, Logistic Regression, Naïve Bayes, and Classification Tree also demonstrated high predictive capability, with AUC values ranging from 0.782 to 0.883.

Figure 7 presents the ROC curves for eight machine learning models trained on the imputed and SMOTE-balanced dataset. The ensemble models, XGBoost and Random Forest, show the best predictive performance with AUC values of 0.891 and 0.885, respectively, and curves that lie closest to the top-left corner of the plot.

**Figure 8:**
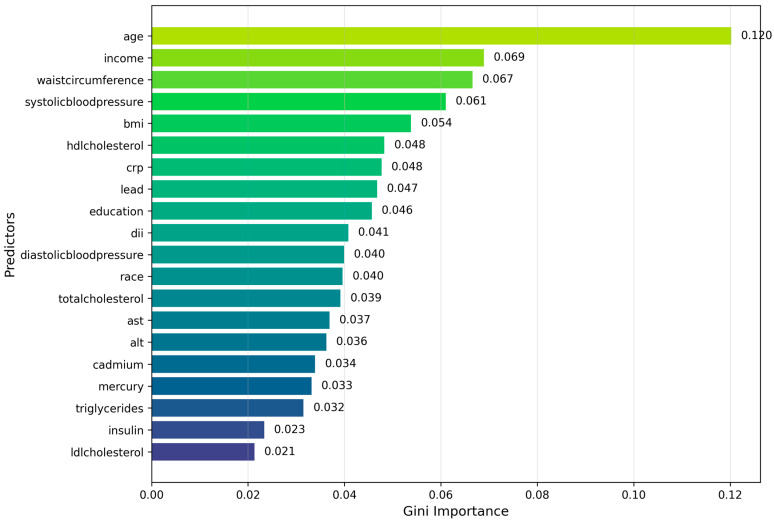
Variable importance rankings derived from the Random Forest model for predicting diagnosed diabetes. Age and income are the most influential predictors. Among environmental exposures, lead contributes the most to model importance.

Additionally, we used a Random Forest model to evaluate predictors contributing most strongly to diabetes classification (Figure 8). The results indicated that age, income, and waist circumference were the most influential predictors. Among the toxic environmental exposures, lead exhibited the highest variable importance within the Random Forest model.

### 3.4. External-Style Validation (80/20 Hold-Out Test)

**Figure 9:**
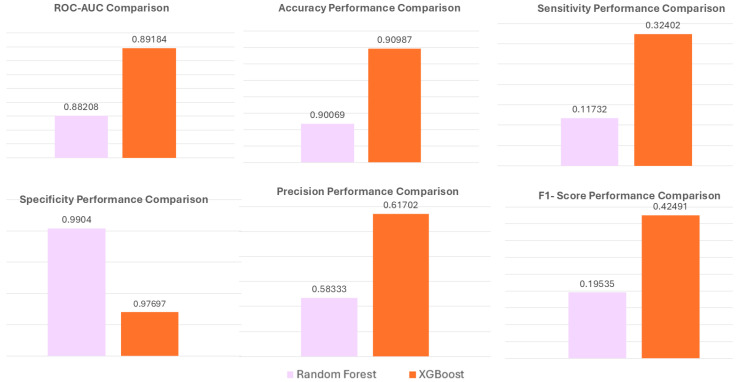
Performance comparison between Random Forest and XGBoost on the independent 20% hold-out test set.

To test how well the results would generalize, we ran an independent validation. We split the fully imputed dataset into 80% for training and 20% for testing, applying preprocessing only to the training data. Both Random Forest and XGBoost were retrained on the training set and then tested on the untouched 20% hold-out set. XGBoost reached an AUC of 0.89184, accuracy of 0.90987, sensitivity of 0.32402, specificity of 0.9904, and an F1 score of 0.42491. These results show that ensemble tree-based models kept their strong performance on new data (Figure 9). These results are consistent with cross-validation outputs and support conclusions regarding model stability and generalizability.

#### Performance on the Independent Hold-Out Test Set

**Figure 10:**
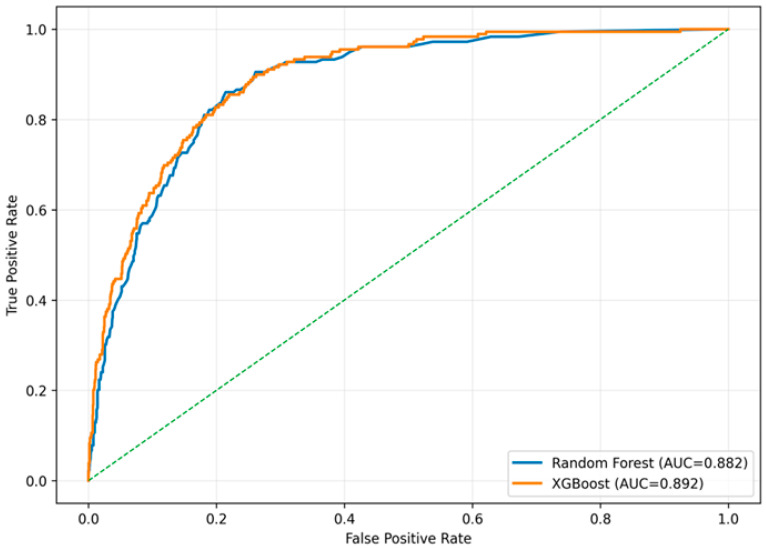
Receiver operating characteristic (ROC) curves for Random Forest and XGBoost, evaluated on the independent 20% hold-out test set. Both models demonstrate strong discrimination, with XGBoost achieving an AUC of 0.892 and Random Forest an AUC of 0.882.

To assess how well the models perform on new data, we conducted an internal validation using a stratified 80/20 train-test split. Preprocessing steps were applied only to the training set to avoid leakage. The ROC curves in Figure 10 show that both XGBoost and Random Forest maintained strong discrimination when evaluated on the hold-out test set. XGBoost achieved an AUC of 0.892, while Random Forest reached an AUC of 0.882. The curves indicate that each model separates cases from non-cases with high accuracy, confirming that the predictive patterns learned during training generalize well to unseen data.

## 4. Discussion

Our approach incorporated multiple domains of information, including demographic, anthropometric, biochemical, behavioral, and environmental exposures. After handling the high rate of missing data with MICE and addressing class imbalance with SMOTE, most machine learning models achieved excellent performance. XGBoost and Random Forest achieved the highest overall discrimination and accuracy, as reflected by their ROC–AUC and accuracy values. These results agree with earlier studies that highlight the strong performance of ensemble tree-based models in clinical prediction and high-dimensional health data analysis [14,15,16].

The variable importance patterns suggested that age, income, and waist circumference were the most important factors. These results are consistent with established life-course and cardiometabolic frameworks in which cumulative metabolic burden, central adiposity, and socioeconomic position are strongly associated with diabetes risk. Measures of blood pressure, lipid metabolism, and systemic inflammation also ranked highly, consistent with cardiometabolic dysregulation serving as an important correlate of diagnosed diabetes. Notably, blood concentrations of lead, cadmium, and mercury contributed predictive information comparable to several traditional clinical risk factors, suggesting that environmental metal exposures may function as informative risk markers that contribute to diabetes classification beyond traditional clinical predictors. Dietary inflammatory potential and educational attainment showed moderate importance, consistent with their roles as behavioral and social modifiers of metabolic risk. Overall, the observed importance structure is consistent with a multifactorial pattern of diabetes classification in which social, metabolic, and environmental factors jointly contribute predictive information.

A key contribution of this study is the integration of environmental exposure biomarkers into diabetes classification models. Metals such as lead contributed meaningfully to model performance. This finding is consistent with prior experimental and epidemiologic studies reporting that low-to-moderate chronic exposure to heavy metals may impair β-cell function, reduce insulin secretion, and increase oxidative stress, processes that have been linked to diabetes risk [42]. The ability of ensemble machine learning models to capture non-linear and higher-order interactions may explain why metals appear influential despite their minimal pairwise correlations.

In contrast, demographic and behavioral variables such as sex, race/ethnicity, income, education, smoking, and alcohol use showed relatively lower importance once clinical and biochemical predictors were included, potentially reflecting their indirect influence through biological pathways and healthcare access. Prior work also suggests that these distal determinants act mainly through proximal biological pathways such as adiposity, lipid levels, and inflammation, rather than directly influencing disease risk. Additionally, the broad categorical measurement of behavioral and socioeconomic variables in NHANES may limit their predictive resolution compared to continuous biomarkers.

A major aspect of this analysis is its meticulous handling of missing data. Several biochemical and environmental variables exhibited severe missingness (60–70%). It makes complete-case analysis infeasible. Simulation experiments show that the use of Multiple Imputation by Chained Equations (MICE) can minimize bias and maintain statistical power in datasets with mixed variable types and high missingness rates [34,35]. Furthermore, the choice of 70 imputations complies with accepted guidelines, which state that to guarantee stable estimates and a suitable representation of imputation uncertainty, the number of imputations should roughly correspond to the percentage of incomplete cases [37].

Addressing the strong class imbalance with SMOTE further ensured that the minority diabetic class was well represented during model training, thereby preventing bias in classification performance [43]. An independent internal 80/20 hold-out validation was performed. The results indicate that both Random Forest and XGBoost maintained strong performance on unseen data.

However, several limitations must be considered. First, diabetes was defined by self-reported physician diagnosis, which may be affected by underdiagnosis, recall errors, and differential access to healthcare, potentially introducing diagnostic and socioeconomic bias [44]. Second, the cross-sectional design of NHANES prevents the assessment of temporality or causal inference, thereby allowing for reverse causation. Third, exposures such as PFAS and heavy metals were examined only at a single time point, which may not correctly represent long-term or cumulative exposure patterns.

Subgroup-specific performance by sex, race or ethnicity, and socioeconomic status was not evaluated in this study, limiting formal assessment of algorithmic fairness. While stratified analyses could provide insight into heterogeneity in model performance and feature contributions, effective sample sizes within subgroups were substantially reduced after multiple imputation and class-imbalance correction. Conducting subgroup-level evaluations under these conditions may produce unstable or unreliable estimates, and was therefore deferred to future work designed specifically for fairness and subgroup robustness analyses.

## 5. Conclusions

In summary, this study found that integrating environmental exposure biomarkers with clinical, metabolic, anthropometric, behavioral, and demographic variables can markedly improve the classification of diagnosed diabetes in NHANES. After applying MICE and SMOTE, Random Forest and XGBoost achieved very high discrimination.

Notably, heavy metal exposures such as lead contributed meaningfully to model performance, indicating that toxicant biomarkers provide additional discriminatory information in diabetes classification. Importantly, the models also performed well on a separate 20% test set. This result indicates that the models generalized beyond the training data and did not simply overfit the development sample.

These results support incorporating environmental exposure biomarkers into population-level diabetes classification models. These combined models can capture complex, non-linear patterns that may not be fully represented in traditional linear modeling approaches. In public health, such models may support population-level diabetes classification, improve risk stratification, and help identify groups that may benefit from targeted metabolic management and exposure-reduction strategies.

## Figures and Tables

**Table 1:** Descriptive statistics for critical variables of the study participants.

Variable	N	Percent
Male	4557	49.24%
Female	4697	50.76%
Race/Hispanic Origin		
Mexican American	1367	14.77%
Other Hispanic	820	8.86%
Non-Hispanic White	3150	34.04%
Non-Hispanic Black	2115	22.85%
Other Race—Including Multi-Racial	634	6.85%
Income		
$0–$4999	282	4.48%
$5000–$9999	252	4.00%
$10,000–$14,999	399	6.33%
$15,000–$19,999	536	8.51%
$20,000–$24,999	529	8.40%
$25,000–$34,999	960	15.24%
$35,000–$44,999	893	14.17%
$45,000–$54,999	607	9.63%
$55,000–$64,999	573	9.09%
$65,000 = $74,999	441	7.00%
$75,000–$99,999	829	13.16%
Education		
Less than 9th Grade	480	8.64%
9–11th grade (Includes 12th grade with no diploma)	639	11.50%
High school graduate/GED or equivalent	1325	23.84%
Some college or AA degree	1778	31.99%
College graduate or above	1336	24.04%
Had at least 12 alcoholic drinks in 1 year		
Yes	4545	88.60%
No	585	11.40%
Smoked at least more than 100 cigarettes in life		
Yes	2359	40.28%
No	3497	59.72%

**Table 2:** Model performance comparison after multiple imputation (M = 70) and SMOTE balancing.

Model	ROC-AUC	Accuracy	Sensitivity	Specificity	Precision	F1 Score
XGBoost	0.89098	0.90573	0.30683	0.97416	0.57563	0.40029
Random Forest	0.88488	0.90079	0.11534	0.99053	0.58192	0.19252
Logistic Reg.	0.88278	0.77965	0.85218	0.77137	0.29867	0.44231
SVM (RBF)	0.86428	0.89528	0.16797	0.97838	0.47022	0.24753
KNN	0.82684	0.90206	0.08959	0.99488	0.66667	0.15795
Naive Bayes	0.82283	0.84373	0.36618	0.89829	0.29144	0.32457
ANN (MLP)	0.78222	0.8745	0.36506	0.9327	0.38263	0.37364
Decision Tree	0.61276	0.86141	0.30011	0.92554	0.31529	0.30752

## Data Availability

The data presented in this study are openly available on the CDC NHANES site at https://wwwn.cdc.gov/nchs/nhanes/continuousnhanes/overview.aspx?BeginYear=2017 (accessed on 1 December 2025).

## Abbreviations

The following abbreviations are used in this manuscript:

NHANES	National Health and Nutrition Examination Survey
CDC	Centers for Disease Control and Prevention
NCHS	National Center for Health Statistics
BMI	Body Mass Index
BP	Blood Pressure
HbA1c	Hemoglobin A1c
PFAS	Per- and Polyfluoroalkyl Substances
PFOA	Perfluorooctanoic Acid
PFOS	Perfluorooctane Sulfonate
Pb	Lead
Cd	Cadmium
Hg	Mercury
LOD	Limit of Detection
MICE	Multiple Imputation by Chained Equations
SMOTE	Synthetic Minority Oversampling Technique
ROC–AUC	Receiver Operating Characteristic—Area Under the Curve
ML	Machine Learning
SVM	Support Vector Machine
ANN	Artificial Neural Network
k-NN	k-Nearest Neighbors
CRP	C-reactive protein
HDL	High-Density Lipoprotein
LDL	Low-Density Lipoprotein
AST	Aspartate Aminotransferase
ALT	Alanine Aminotransferase
SEQN	Survey Participant Identifier
